# Epidemiologic relationship between alcohol flushing and smoking in the Korean population: the Korea National Health and Nutrition Examination Survey

**DOI:** 10.1038/s41598-024-66521-z

**Published:** 2024-07-08

**Authors:** Hwa Jung Yook, Gyu-Na Lee, Ji Hyun Lee, Kyungdo Han, Young Min Park

**Affiliations:** 1 Department of Dermatology, Seoul St. Mary’s Hospital, College of Medicine, The Catholic University of Korea, 222 Banpo-daero, Seocho-gu, Seoul, 06591 Korea; 2 Department of Biomedicine & Health Science, The Catholic University of Korea, Seoul, Korea; 3 Department of Statistics and Actuarial Science, Soongsil University, Seoul, Korea

**Keywords:** Epidemiology, Patient education

## Abstract

Although facial flushing after drinking alcohol (alcohol flushing response) is common in Asian populations, the epidemiological features in a large sample have been investigated in only a few studies. This study assessed the epidemiologic characteristics and associated factors for alcohol flushing in a Korean population. This study was based on data collected during the 2019 Korea National Health and Nutrition Examination Survey (KNHANES). A total of 5572 Korean adults was included in the general population group, and the alcohol flushing group consisted of 2257 participants. Smoking and physical activity were evaluated as possible associated factors for alcohol flushing. The overall prevalence of alcohol flushing was estimated at 40.56% of the general population (43.74% in males and 37.4% in females), and the prevalence was highest at 60–69 years of age and lowest in individuals older than 80 years. Occasional, frequent, and persistent alcohol flushing was reported by 11.9%, 3.7% and 15.0% of current flushers, among whom persistent flushers consumed the least amount of alcohol. Subjects who currently smoke had a higher propensity of alcohol flushing (adjusted OR 1.525, 95% CI 1.2–1.938), and subjects with smoking history of 20–29 pack-years (PYs) showed the highest association (adjusted OR 1.725, 95% CI 1.266–2.349) with alcohol flushing after adjustment for confounders. In contrast, significant association was not found between physical activity and alcohol flushing. The results demonstrated that current smoking status is shown to be significantly associated with alcohol flushing, and that current smokers with a history of smoking ≥ 20 PYs had a higher likelihood of alcohol flushing than non-smokers or ex-smokers.

## Introduction

Flushing refers to temporary reddening of the skin, particularly on the face, neck, upper chest, and other areas, accompanied by a feeling of warmth^1,2^. Flushing and blushing are caused by physiological transient cutaneous vasodilatation. Although these terms are often used interchangeably, a blush represents a psychosocial response to an emotion, whereas a flush is due to a thermoregulatory response to elevated body temperature^2^. Alcohol flushing response, known as drinking-related flushing, is mainly caused by the accumulation of acetaldehyde caused by alcohol metabolism^3^, and acetaldehyde is then metabolized to acetate by aldehyde dehydrogenase enzymes, mainly aldehyde dehydrogenase 2 (ALDH2)^4,5^. Alcohol flushing and ALDH2 gene polymorphism are common among East Asian populations including Japanese, Chinese, and Korean^6^, and the term “Asian flush syndrome” or “Oriental flushing” have been used to describe facial flushing, headache, nausea, dizziness, and cardiac palpitations after consumption of alcoholic beverages^2^. Therefore, facial flushing after alcohol intake is regarded as a predictor of inactive ALDH2^5,7^. The point mutation in ALDH2, identified as ALDH2∗2, is present in 8% of the world’s population, or approximately 560 million people. ALDH2 ∗ 2 variant incidence in East Asian ethnicities ranged from 28 to 54%^8^. In previous epidemiologic studies including Japanese subjects, questionnaires concerning alcohol flushing as a surrogate marker of inactive ALDH2 genotype have been used^9–11^.

To date, the primary focus in previous research was on comorbidities associated with alcohol flushing. Kim et al.^12^ suggested that some Korean male drinkers who experience an alcohol flushing response have a higher risk of metabolic syndrome and hypertension with less alcohol consumption than non-flushers^13^. In addition, flushers were reported to have an increased risk of coronary spastic angina as well as esophageal, pharyngolaryngeal, and bladder cancer^5,6,14–16^. Yokoyama et al.^17^ reported that never or former flushing and genotype combinations were independent strong risk factors of alcohol dependence in Japanese men and women. While the clinical importance of an alcohol flushing response is emphasized in various medical fields^5,13,16,18^, the epidemiology and associated factors for alcohol flushing have not been sufficiently investigated. This study aims to investigate the epidemiologic characteristics of alcohol flushing and to identify the possible associated factors for alcohol flushing in the Korean population using data from the Korea National Health and Nutrition Examination Survey (KNHANES). Furthermore, subgroup analyses stratified based on drinking and flushing status were performed to clarify the association between the two variables.

## Results

### Epidemiologic characteristics and prevalence of alcohol flushing

**Figure 1:**
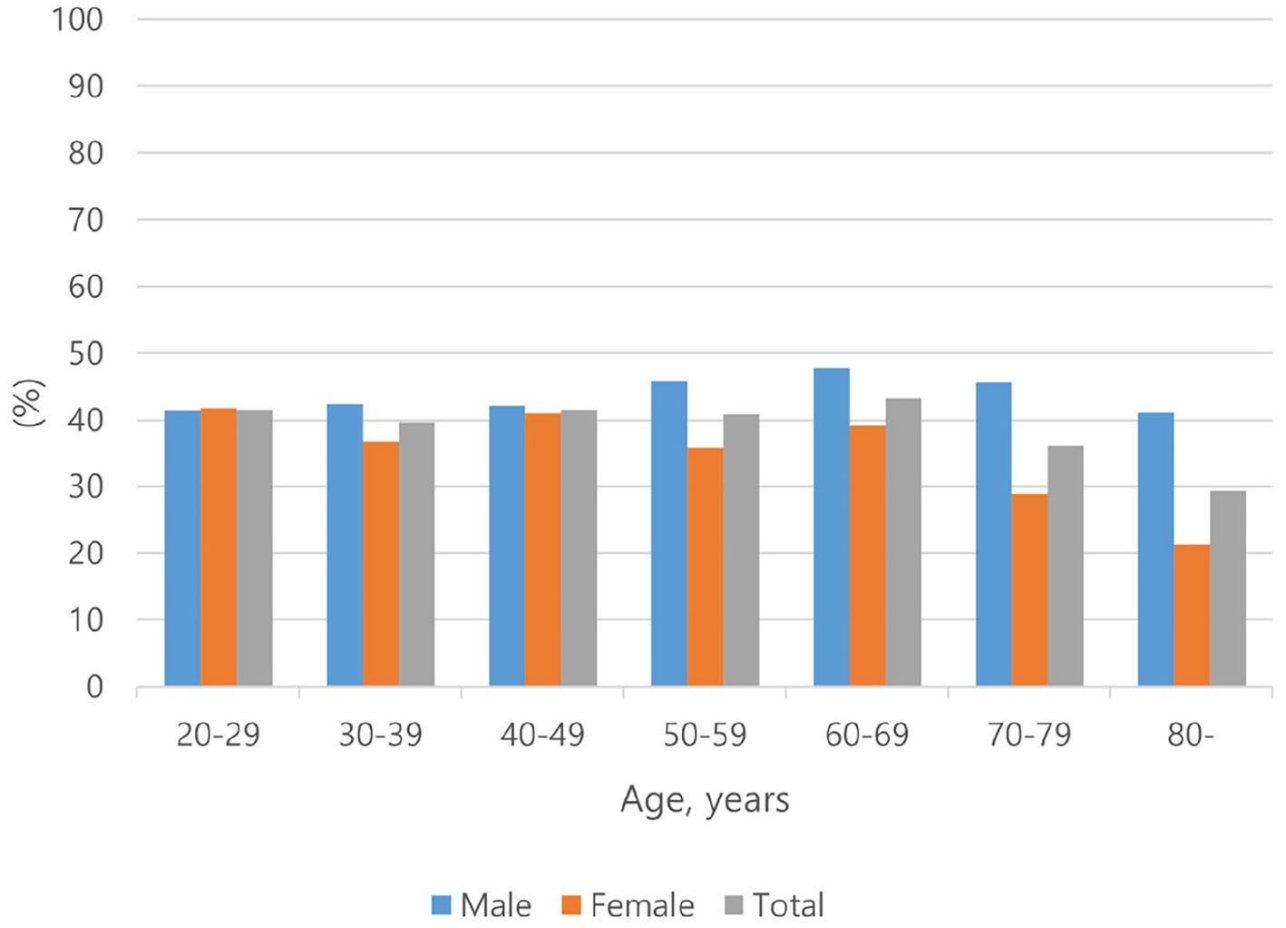
Prevalence of alcohol flushing based on age and sex.

Table 1 shows the demographic characteristics of the study population based on drinking and alcohol flushing. The age- and sex-standardized prevalence of alcohol flushing is shown in Fig. 1. The overall prevalence of alcohol flushing was estimated at 40.56% of the general population (43.74% in males and 37.4% in females), and the prevalence was highest when subjects were 60–69 years of age and lowest in individuals > 80 years of age.

**Table 1:** General characteristics of the study population based on drinking and alcohol flushing status (n = 5572).

n	Non-drinker	Drinker		p-value^a^	p-value^b^
	577	Non-flusher	Flusher		
		2738	2257		
Age (years)	61.85 (1.1)	45.37 (0.45)	46.96 (0.49)	< 0.0001	0.0005
Sex					
Male	22.91 (2.18)	51.08 (1.08)	53.72 (1.07)	< 0.0001	0.1225
Female	77.09 (2.18)	48.92 (1.08)	46.28 (1.07)		
Low income	35.04 (2.61)	11.77 (0.83)	13.54 (1.02)	< 0.0001	0.0825
High educational level	49.57 (2.81)	84.26 (1.02)	81.57 (1.24)	< 0.0001	0.0115
Smoking status					
Non-smoker	87.06 (1.77)	58.31 (1.22)	53.98 (1.26)	< 0.0001	0.0213
Ex-smoker	7.12 (1.29)	21.99 (0.92)	22.99 (0.97)		
Current smoker	5.82 (1.47)	19.7 (0.97)	23.03 (1.21)		
Physical activity	36.78 (2.45)	47.64 (1.32)	45.79 (1.2)	0.0005	0.2462
Diabetes mellitus	21.01 (1.92)	10.55 (0.66)	11.95 (0.82)	< 0.0001	0.1361
Hypertension	47.53 (2.58)	24.77 (1.1)	26.01 (1.26)	< 0.0001	0.3838
Hypercholesterolemia	31.61 (2.37)	21.31 (0.97)	20.52 (0.97)	< 0.0001	0.5237
BMI	24 (0.17)	23.93 (0.1)	23.91 (0.09)	0.8883	0.849
Waist circumference	84.19 (0.5)	83.48 (0.29)	83.81 (0.28)	0.4304	0.4272
Systolic BP	125.34 (0.94)	117.14 (0.39)	117.74 (0.44)	< 0.0001	0.249
Diastolic BP	74.41 (0.58)	76.32 (0.26)	75.94 (0.3)	0.0071	0.2804
Serum glucose	103.01 (1.08)	99.44 (0.47)	100.18 (0.71)	0.0095	0.3533
Total cholesterol	189.08 (1.93)	194.25 (0.82)	192.46 (0.94)	0.0342	0.1608
Serum HDL	50.93 ± 0.6)	53.9 (0.34)	52.04 (0.34)	< 0.0001	< 0.0001

Data are expressed as the mean (SD) or n (%).

*BP* blood pressure, *SD* standard deviation, *BMI* body mass index, *HDL* high-density lipoprotein.

^a^indicates comparison of three groups: non-drinker, non-flusher, and flusher.

^b^indicates comparison between two groups: non-flusher and flusher.

**Figure 2:**
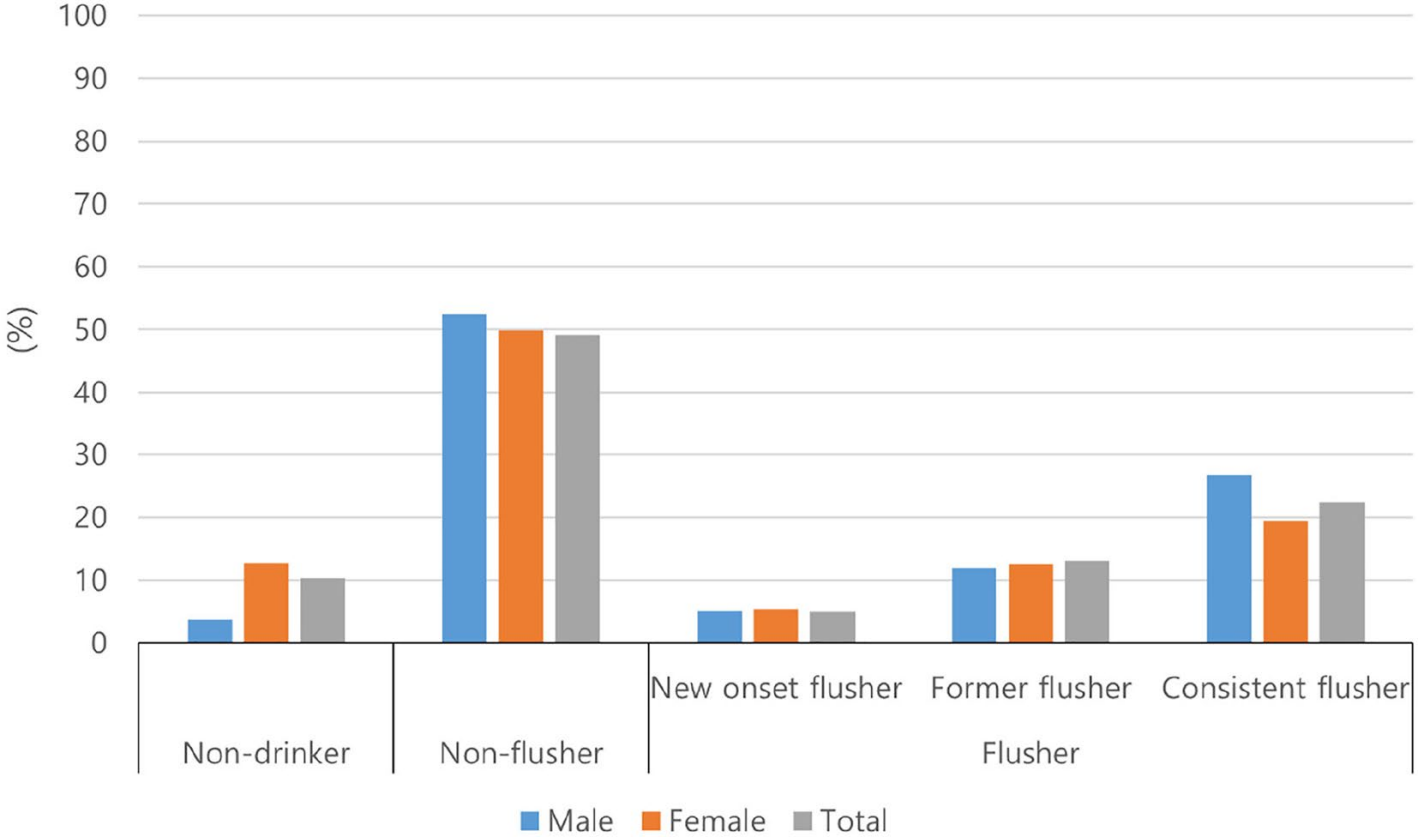
Past and current alcohol flushing status.

In male and female subjects, 3.81% (n = 108) and 12.73% (n = 469) were non-drinkers, 52.45% (n = 1265) and 49.87% (n = 1473) were non-flushers, 5.04% (n = 121) and 5.29% (n = 155) were new-onset flushers, 11.94% (n = 323) and 12.66% (n = 408) were former flushers, and 26.76% (n = 655) and 19.45% (n = 595) were consistent flushers, respectively (Fig. 2). Collectively, 2738 (54.8%) and 731 (14.6%) individuals were non-flushers and former flushers, respectively. Among 1526 (30.6%) current flushers, 593 (11.9%), 183 (3.7%), and 750 (15.0%) participants reported having alcohol flushing responses occasionally, frequently, and always, respectively. To determine whether the drinking amount differed based on flushing group, more segmented ranges of the amount of alcohol consumed were measured. Among the current-occasional flushers, 71.5%, 7.4%, and 21.1% consumed < 10 g per day, 10–20 g per day, and > 30 g per day of alcohol, respectively. Among the current-frequent flushers, 85.4%, 6.4%, and 8.2% consumed < 10 g per day, 10–20 g per day, and > 30 g per day of alcohol, respectively. In addition, among current-always flushers, 92.6%, 4.1%, and 3.3% consumed < 10 g per day, 10–20 g per day, and > 30 g per day of alcohol, respectively. These results demonstrated the lowest alcohol consumption in the current-always group (Fig. S1).

### Smoking and physical activity as associated factors for alcohol flushing

Tables 2 and 3 shows the ORs of any experience of alcohol flushing and current status of alcohol flushing based on smoking status, respectively. In Table 2, for current smokers, the OR of alcohol flushing was 1.556 (95% CI 1.235–1.96, model 3), and for subjects with 20–29 years of PYs, the OR was 1.796 (95% CI 1.343–2.402, model 3). The odds of experiencing alcohol flushing were 91.3% higher in the current smokers with ≥ 20 PYs than in the non-smokers (95% CI 1.38–2.654 for a combination of smoking status and PYs, model 3).

**Table 2:** Any experience of alcohol flushing based on smoking status and/or PYs in multivariate logistic regression.

	Adjusted OR (95% CI)								
	% (SE)	Crude OR (95% CI)	p-value	Model1	p-value	Model2	p-value	Model3	p-value
Smoking status									
Non-smoker	37.15 (1.05)	1 (ref)	0.0002	1(ref)	0.009	1 (ref)	0.0008	1 (ref)	0.0008
Ex-smoker	42.59 (1.9)	1.255 (1.061–1.485)		1.22 (1.005–1.48)		1.291 (1.061–1.571)		1.29 (1.061–1.57)	
Current smoker	46.41 (2.09)	1.465 (1.217–1.764)		1.395 (1.126–1.73)		1.557 (1.236–1.96)		1.556 (1.235–1.96)	
PYs									
Non	37.15 (1.05)	1 (ref)	< .0001	1 (ref)	0.0008	1 (ref)	0.0001	1 (ref)	0.0001
< 10 PYs	47.34 (2.46)	1.521 (1.233–1.877)		1.422 (1.137–1.78)		1.53 (1.214–1.928)		1.53 (1.214–1.929)	
10–20 PYs	37.27 (2.28)	1.005 (0.812–1.246)		0.964 (0.762–1.22)		1.044 (0.818–1.333)		1.048 (0.821–1.338)	
20–30 PYs	49.72 (3.05)	1.673 (1.306–2.143)		1.641 (1.236–2.177)		1.808 (1.351–2.421)		1.796 (1.343–2.402)	
≥ 30 PYs	45.21 (3.73)	1.396 (1.028–1.897)		1.373 (0.96–1.963)		1.549 (1.08–2.221)		1.54 (1.074–2.209)	
Smoking status in PYs									
Non-smoker	37.15 (1.05)	1 (ref)	< .0001	1 (ref)	0.0005	1 (ref)	< .0001	1 (ref)	< .0001
Ex-smoker, < 20 PYs	39.97 (2.48)	1.126 (0.909–1.396)		1.094 (0.872–1.373)		1.143 (0.91–1.435)		1.143 (0.91–1.434)	
Ex-smoker, ≥ 20 PYs	48.21 (2.19)	1.575 (1.304–1.902)		1.632 (1.277–2.085)		1.754 (1.371–2.243)		1.751 (1.369–2.241)	
Current smoker, < 20 PYs	45.61 (2.38)	1.419 (1.147–1.756)		1.335 (1.058–1.685)		1.473 (1.155–1.877)		1.475 (1.156–1.883)	
Current smoker, ≥ 20 PYs	49.62 (3.28)	1.667 (1.278–2.175)		1.695 (1.238–2.323)		1.928 (1.391–2.673)		1.913 (1.38–2.654)	

Model 1 is adjusted for age and sex.

Model 2 is adjusted for Model 2 + low income, heavy drinking and regular physical activity.

Model 3 is adjusted for Model 3 + presence of hypertension, DM and hypercholesterolemia.

*PYs* pack-years, *SE* standard error, *OR* odds ratio, *CI* confidence interval.

**Table 3:** Current status of alcohol flushing based on smoking status and/or PYs in multivariate logistic regression.

	Adjusted OR (95% CI)								
	% (SE)	Crude OR (95% CI)	p-value	Model 1	p-value	Model 2	p-value	Model 3	p-value
Smoking status									
Non-smoker	25.25 (0.9)	1 (ref)	< 0.0001	1 (ref)	0.0089	1 (ref)	0.0015	1 (ref)	0.0015
Ex-smoker	31.11 (1.86)	1.336 (1.108–1.612)		1.27 (1.026–1.572)		1.323 (1.073–1.632)		1.325 (1.076–1.631)	
Current smoker	35.5 (1.91)	1.629 (1.354–1.96)		1.398 (1.12–1.745)		1.529 (1.204–1.943)		1.525 (1.2–1.938)	
PYs									
Non	25.25 (0.9)	1 (ref)	< 0.0001	1 (ref)	0.0027	1 (ref)	0.0007	1 (ref)	0.0008
< 10 PYs	38.62 (2.35)	1.863 (1.502–2.31)		1.469 (1.164–1.854)		1.548 (1.219–1.965)		1.549 (1.221–1.965)	
10–20 PYs	28.36 (2.04)	1.172 (0.949–1.447)		1.063 (0.839–1.347)		1.135 (0.888–1.45)		1.144 (0.896–1.46)	
20–30 PYs	35.01 (2.82)	1.594 (1.229–2.068)		1.613 (1.187–2.193)		1.751 (1.283–2.389)		1.725 (1.266–2.349)	
≥ 30 PYs	26.35 (3.26)	1.059 (0.758–1.479)		1.096 (0.736–1.63)		1.207 (0.811–1.797)		1.187 (0.797–1.768)	
Smoking status in PYs									
Non-smoker	25.25 (0.9)	1 (ref)	< 0.0001	1 (ref)	0.0268	1 (ref)	0.005	1 (ref)	0.0058
Ex-smoker, < 20 PYs	31.04 (2.3)	1.332 (1.063–1.67)		1.201 (0.942–1.531)		1.234 (0.971–1.569)		1.238 (0.974–1.572)	
Ex-smoker, ≥ 20 PYs	28.79 (2.05)	1.197 (0.978–1.464)		1.356 (1.042–1.766)		1.44 (1.109–1.869)		1.434 (1.106–1.859)	
Current smoker, < 20 PYs	36.91 (2.24)	1.732 (1.403–2.137)		1.392 (1.102–1.759)		1.509 (1.178–1.932)		1.513 (1.18–1.94)	
Current smoker, ≥ 20 PYs	32.2 (3.1)	1.406 (1.056–1.872)		1.496 (1.059–2.113)		1.665 (1.168–2.372)		1.623 (1.138–2.315)	

Model 1 adjusted for age and sex.

Model 2 adjusted for Model 1 + low income, heavy drinking, and regular physical activity.

Model 3 adjusted for Model 2 + presence of hypertension, diabetes mellitus, and hypercholesterolemia.

In Table 3, before adjustment, for current smokers, the OR was 1.629 (95% CI 1.354–1.96, model 1). When adjusting for variables, for current smokers, the OR was 1.525 (95% CI 1.2–1.938, model 3). In particular, for subjects with 20–29 PYs, the OR was 1.725 (95% CI 1.266–2.349, model 3). The odds of the current alcohol flushing were 62.3% higher in the current smokers with ≥ 20 PYs than in the non-smokers (95% CI 1.138–2.315 for a combination of smoking status and PYs, model 3). Notably, among subjects within the same category of PYs, current smokers had a higher OR for alcohol flushing than ex-smokers.

The OR for current alcohol flushing during different types of physical activity was analyzed based on age- and sex-specific groups as shown in Table S1. After adjustment for household income, drinking amount, hypertension, DM, and hypercholesterolemia, discernible differences were not observed in the ORs. Although the unadjusted analysis showed a significant positive relationship between aerobic exercise and the prevalence of alcohol flushing in current alcohol flushers (OR 1.175, 95% CI 1.02–1.353, p = 0.0259), this relationship was lost after adjusting for multiple variables. Similarly, a significant relationship was not observed between the prevalence of alcohol flushing and other physical activities such as walking and strength training.

## Discussion

Alcohol flushing affects all ethnic groups, and the estimated prevalence of alcohol flushing is 2–29% in Caucasians, from 10 to 80% in Native Americans, and relatively higher in East Asians than in other ethnic groups^19^. In a study using Korean Community Health Survey data, the prevalence was 34.8%^20^; alcohol flushing was shown to affect approximately 36–50% of East Asians (Koreans, Chinese, and Japanese) in previous studies^14,19,21^. In the present study using a nationwide population-based data, the mean annual prevalence of alcohol flushing was 40.56%, similar to previous reports. The high prevalence of alcohol flushing among East Asians has been explained in the literature. Multiple studies reported that flushing responses after drinking a small amount of alcohol were mainly attributable to high acetaldehyde exposure and influenced by ALDH2, ADH2^7,10,22–24^ and ADH1B^17^ genotypes. Thus, this phenomenon is predominantly due to an inherited deficiency in the ALDH2 enzyme, and genetic factors associated with discrepancies in ALDH2 and other haplotypes between ethnic groups might lead to differences in prevalence^2^. When measured based on sex and age, the prevalence of alcohol flushing was higher in males, which was consistent with a previous study^20^. Notably, in the present study, 11.94% of males and 12.66% of females were former flushers, and 5.04% of males and 5.29% of females transitioned into new-onset flushers. Jeon et al.^20^ reported that, among participants in the Korean Community Health Survey, 4.1% were former flushers and 34.8% were current flushers. According to Yokoyama et al.^25^, alcohol flushing decreases in intensity due to the development of tolerance to acetaldehyde in blood by higher-risk persons with a long or heavy drinking history. Acquired etiologies have been reported to include use of topical tacrolimus or liver-metabolized drugs, which can aid in alcohol catabolism^26,27^.

In the present study, smoking and physical activity were investigated as possible factors associated with alcohol flushing because they previously showed a statistical demographic difference among flushers, non-flushers, and non-drinkers in our data. We had considered individual alcohol consumption patterns and the efficiency of alcohol metabolism other than genetic factors to elucidate the phenomenon of alcohol flushing. Demographic factors such as age, education level, monthly income and health-related behaviors were documented factors associated with alcohol use in the literature, while underlying diseases and overall health status were considered from the perspective of alcohol metabolism. Therefore, we included these various factors as covariates for adjustment. Consequently, smoking was strongly associated with alcohol flushing regardless of sex. Conversely, significant association was not found between alcohol flushing and physical activity. Furthermore, current smoking is associated with an increase in alcohol flushing. Specifically, the likelihood of alcohol flushing was highest in current smokers with a smoking history ≥ 20 PYs. Although the adjusted OR for alcohol flushing did not proportionally correlate with PYs, further analysis showed a similar trend of higher alcohol flushing propensity as smoking duration increased. In addition, current smokers with the same PYs had a higher OR for alcohol flushing than ex-smokers. Therefore, smoking status possibly has a greater effect on alcohol flushing than the duration of smoking. Moreover, these results demonstrated similar trends in both “any experience of flushing” and “current status of flushing”. In “any experience of flushing (Table 2)”, former flushers were classified as flushers for statistical analysis, whereas in “current status of alcohol flushing (Table 3)”, former flushers were classified as non-flushers. This categorization is based on their current non-flushing status, even though they might have experienced flushing with small amounts of alcohol at the beginning of their drinking history, likely indicating possession of the inactive ALDH2 genotype^10^. This approach demonstrated the significance of separately analyzing former flushers, as their unique transition from flusher to non-flusher status might provide insights into the impact of smoking on alcohol flushing. These consistent tendencies in both “any experience of flushing” and “current status of flushing” can further support the association between smoking and alcohol flushing status. To the best of our knowledge, this is the first study in which a positive relationship between smoking and alcohol flushing was demonstrated; however, this correlation may have been postulated in other research. Reportedly, acetaldehyde can produce angiogenesis and telangiectasia through the expression of vascular endothelial growth factor (VEGF), as experimentally demonstrated in the chick embryo chorioallantoic membrane model^28^, and nicotine provokes dry flushing via vasodilation action of prostaglandins on vascular smooth muscle^29^. El-Zayadi et al.^30^ proposed that the direct action of vasodilator constituents in smoke, along with excess hemoglobin, serves as mechanisms for the facial flushing observed in heavy smokers.

Flushers may have excessive acetaldehyde accumulation with low alcohol consumption, and such mechanisms could affect these individuals after minimal alcohol use^14,31^. Further biological research is needed to fully understand this relationship.

However, the results of the present study revealed that individuals who experience alcohol flushing usually consume alcohol less frequently and in smaller quantities compared with subjects who do not experience these symptoms, with most drinking < 10 g of alcohol per day. This observation is in agreement with earlier studies that have shown people with inactive ALDH2 to be more likely to refrain from drinking^20,32^. Considering the pervasive nature of social gatherings and interactions within South Korea’s drinking culture, where smoking and drinking frequently coexist^33^, it is our contention that even though individuals may consume lesser amounts of alcohol due to alcohol flushing, smoking may still play a significant role in triggering alcohol flushing reaction.

Because the focus in the present study was on self-reported alcohol flushing status, the accuracy of alcohol flushing status determined solely through self-reported symptoms compared with genetic testing cannot be ensured. Although the use of genetic variants may provide a more reliable measure, based on the available data, whether the responses obtained for alcohol flushing accurately reflect the deficiency of the alcohol-metabolizing enzyme cannot be determined^34,35^. In addition, although the alcohol flushing response is mainly explained due to an inactive ALDH2, with a sensitivity of 95.1% and specificity of 76.5%^36^, individuals exhibiting this response may have characteristics different from subjects with an active ALDH2. The flushing response can be influenced by factors other than an inactive ALDH2, such as environmental factors or other genetic traits^7,10,17,22–24^. Also, self-reported data may be subject to recall or measurement bias, especially for alcohol-related behaviors, which may be under- or over-reported. Despite these limitations, the relationships were investigated in a nationally representative sample of Koreans, producing sufficient statistical power. Furthermore, relevant confounding factors were considered. Based on our current understanding, this is the first study in which the associated factors for alcohol flushing were evaluated using nationally representative data. In addition, the KNHANES survey includes central laboratory data and a standardized questionnaire administered by a trained examiner.

In conclusion, the results of the present study demonstrated that current smoking is associated with higher odds of the alcohol flushing, and current smokers with > 20 PYs of smoking history have a higher likelihood of alcohol flushing than non-smokers or ex-smokers.

## Methods

### Data source and study population

**Figure 3:**
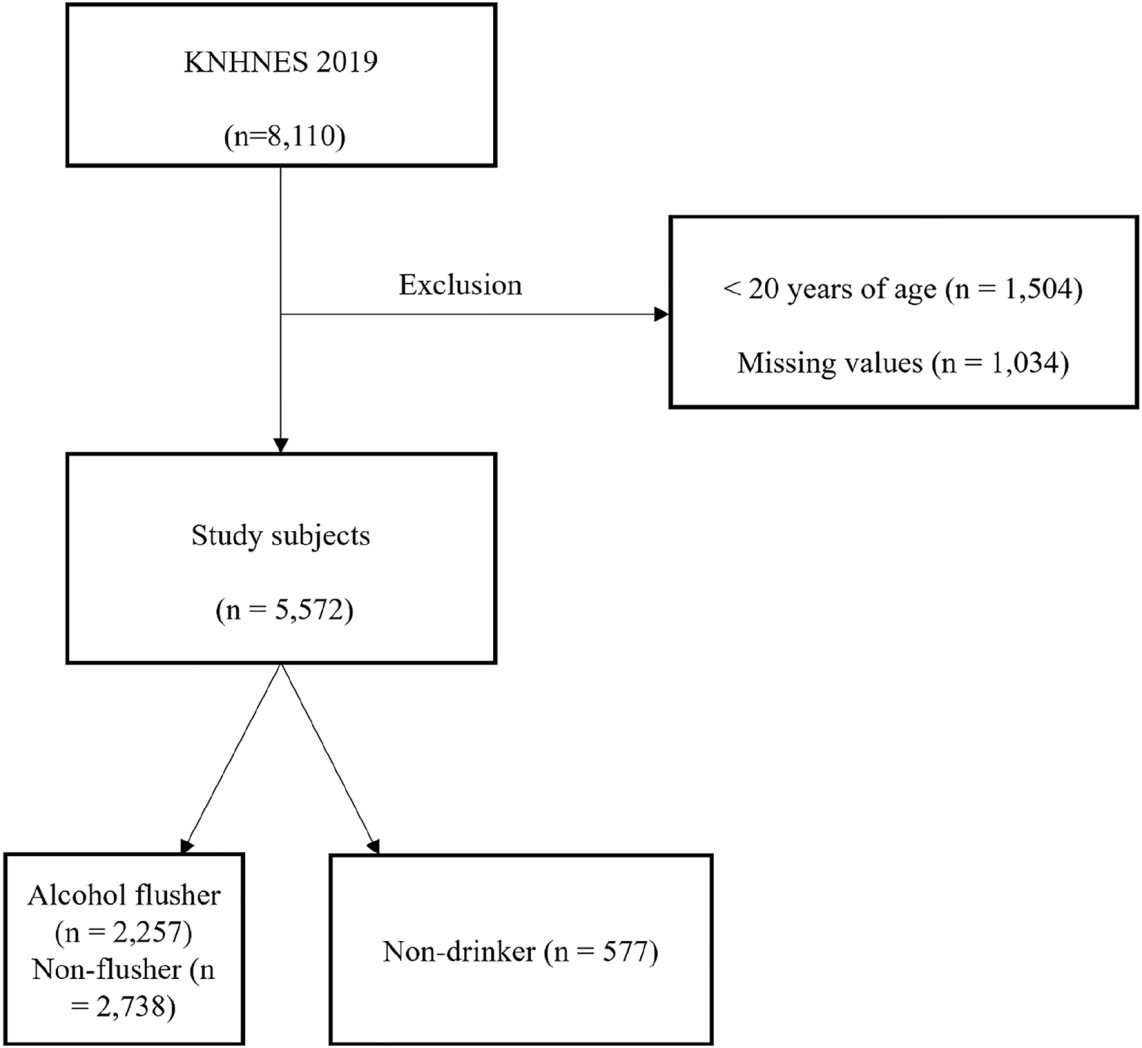
Flow chart of study subject selection. *KNHANES* Korea National Health and Nutrition Examination Survey.

This study included data collected during the 2019 KNHANES, a survey designed to accurately assess national health and nutrition levels and a nationwide study of non‑institutionalized civilians that uses a stratified and multi‑stage probability sampling design with a rolling survey‑sampling model. A total of 8110 participants completed this survey. Individuals < 20 years of age were excluded because this age group is not allowed to drink. After further exclusion of subjects with missing data on alcohol flushing or lifestyle, comorbidities, and laboratory results, 5572 participants were available for analysis (2472 males and 3100 females; Fig. 3). The participants were stratified based on sex and age. A detailed description of the plan and operation of the survey is available on the KNHANES website (https://knhanes.kdca.go.kr/knhanes/sub03/sub03_06_02.do).

### Data collection

All participants were questioned regarding their demographic variables, socioeconomic characteristics, and medical history. The subjects also answered questions regarding history of smoking, drinking, and physical activity through a self-administered questionnaire. Smoking status was categorized as current, ex-, or non-smoker. Ex-smokers had smoked in the past but did not smoke at the time of the interview. Period (years) and amount (in terms of packs) of smoking were included in the questions for ex- and current smokers. Pack-years (PYs) smoked were estimated by multiplying average daily cigarette smoking by smoking duration and was categorized as follows: < 10 PYs, 10–20 PYs, 20–30 PYs, or > 30 PYs. In addition, PYs were categorized in 20 year intervals with current smoking status.

Similarly, drinking status was categorized as current drinker, ex-drinker, or non-drinker. Data on frequency and amount of alcohol consumed per day were also collected and categorized based on daily consumption. The low-income category corresponded to the lowest quartile of annual household income. The educational level of the subject was classified as high if the participant had completed 10 years of education. Physical activity (aerobic, walking, and strength training) at the time of survey was also assessed. The participant’s height, weight, and waist circumference were measured. Body mass index (BMI) was calculated by dividing weight in kilograms by height in meters squared (kg/m^2^). Waist circumference was measured parallel to the floor from the iliac crest in the resting position. Blood samples were collected from the antecubital vein of each participant after fasting for > 8 h to measure concentrations of serum fasting plasma glucose, total cholesterol, high-density lipoprotein (HDL) cholesterol, and triglycerides.

### Assessment of alcohol consumption and alcohol flushing

Alcohol consumption was assessed by questioning the subjects regarding their drinking behavior, including the average amount consumed and drinking frequency, in the year before the interview. A standard drink was defined as a single glass of liquor, wine, or the Korean traditional distilled liquor So-ju. One bottle of beer (355 mL) was counted as 1.6 standard drinks. The amount of alcohol consumed per standard drink was calculated as 10 g, and the average daily alcohol intake was assessed. An average consumption ≥ 30 g per day, a level of exposure associated with health risks, was considered heavy alcohol drinking. In the survey, the question on alcohol flushing status was divided into two parts. First, respondents were asked, “During the first 1–2 years when you started drinking alcohol, did you have facial flushing after consuming a small amount of alcohol?” Response options included (1) yes, (2) no, (3) non-applicable (ex. non-drinker), and (4) unknown or no response. Second, respondents were asked, “Do you currently experience facial flushing after consuming a small amount of alcohol?” Response options included (1) no, (2) occasionally, (3) frequently, and (4) always.

Individuals were categorized as “non-flusher” if they never experienced alcohol flushing, “new-onset” if they recently started experiencing alcohol flushing, “former” if they used to experience alcohol flushing but do not currently, and “consistent flusher” if they consistently experienced alcohol flushing in the past and present.

### Statistical analysis

All analyses were conducted using SAS version 9.4 (SAS Institute Inc., Cary, NC, USA) to reflect the complex sampling design and sampling weights of the KNHANES and to provide nationally representative prevalence estimates. The mean ± standard error (SE) for continuous variables or the percentage for categorical variables was calculated. A one-way ANOVA or a Chi-square test was used to compare the groups. Multiple logistic regression analyses were used to estimate the prevalence odds ratio (OR) and 95% confidence interval (CI) of alcohol flushing. Several models were applied to evaluate the potential mediation effects of modifiable behaviors such as drinking or exercise as well as the effects of known risk factors such as comorbidities. Thus, model 1 was adjusted for age and sex; model 2 was further adjusted for household income, heavy drinking, and regular physical activity; model 3 was further adjusted for hypertension, diabetes mellitus (DM), and hypercholesterolemia. p < 0.05 was considered statistically significant.

### Ethics statement

The Institutional Review Board of The Catholic Medical Center approved the study protocol (KC23ZASI0282).

### Supplementary Information

Supplementary Information.

## Data Availability

Sequence data that support the findings of this study have been deposited in https://knhanes.kdca.go.kr/knhanes/sub03/sub03_02_05.do.
