# Supplementary material for: Epidemiologic relationship between alcohol flushing and smoking in the Korean population: the Korea National Health and Nutrition Examination Survey
Source: Sci Rep. 2024 Jul 8;14:15710. doi: 10.1038/s41598-024-66521-z (PMC11231332; doi:10.1038/s41598-024-66521-z)
Supplement: Supplementary file 1 — Supplementary Information. [file 41598_2024_66521_MOESM1_ESM.docx]

**Table S1.** Current status of alcohol flushing based on physical activity in multivariate logistic regression

|  |  | |  |  | | | Adjusted OR (95% CI) | | | | | |
| --- | --- | --- | --- | --- | --- | --- | --- | --- | --- | --- | --- | --- |
|  | % (SE) | Crude OR (95% CI) | | | p-value | Model 1 | | p-value | Model 2 | p-value | Model 3 | p-value |
| Aerobic exercise |  | |  | |  |  | |  |  |  |  |  |
| No | 26.79 (1.08) | | 1 (ref) | | 0.0259 | 1 (ref) | | 0.619 | 1 (ref) | 0.7138 | 1 (ref) | 0.7273 |
| Yes | 30.06 (1.12) | | 1.175 (1.02–1.353) | |  | 1.038 (0.895–1.205) | |  | 1.028 (0.884–1.196) |  | 1.027 (0.883–1.196) |  |
| Walking |  | |  | |  |  | |  |  |  |  |  |
| No | 28.16 (1.06) | | 1(ref) | | 0.7948 | 1 (ref) | | 0.6186 | 1 (ref) | 0.4305 | 1 (ref) | 0.4335 |
| Yes | 28.55 (1.17) | | 1.02 (0.88–1.182) | |  | 0.963 (0.828–1.119) | |  | 0.938 (0.799–1.101) |  | 0.938 (0.799–1.102) |  |
| Strength training |  | |  | |  |  | |  |  |  |  |  |
| No | 27.56 (0.98) | | 1 (ref) | | 0.0902 | 1 (ref) | | 0.917 | 1 (ref) | 0.9436 | 1 (ref) | 0.995 |
| Yes | 30.8 (1.6) | | 1.17 (0.976–1.402) | |  | 1.01 (0.837–1.219) | |  | 1.007 (0.829–1.223) |  | 1.001 (0.822–1.218) |  |

Model 1 adjusted for age and sex.

Model 2 is adjusted for Model 2 + low income, heavy drinking and regular physical activity.

Model 3 is adjusted for Model 3 + presence of hypertension, DM and hypercholesterolemia.

Acronyms: SE, standard error; OR, odds ratio; CI, confidence interval

**Figure S1.** Alcohol consumption amount based on alcohol flushing status
